# Hymenopteran Parasitoids of Aphid Pests within Australian Grain Production Landscapes

**DOI:** 10.3390/insects12010044

**Published:** 2021-01-08

**Authors:** Samantha E. Ward, Paul A. Umina, Sarina Macfadyen, Ary A. Hoffmann

**Affiliations:** 1 Bio21 Institute, School of BioSciences, The University of Melbourne, Parkville, VIC 3010, Australia or pumina@cesaraustralia.com (P.A.U.); ary@unimelb.edu.au (A.A.H.); 2 Cesar Australia, 293 Royal Parade, Parkville, VIC 3052, Australia; 3 CSIRO Entomology, Black Mountain, GPO Box 1700, Canberra, ACT 2601, Australia; Sarina.Macfadyen@csiro.au

**Keywords:** agriculture, grains, Aphididae, Aphidiinae, *Diaeretiella rapae*, natural enemies, biological control, hymenoptera

## Abstract

**Simple Summary:**

In grain crops, aphids are important pests, but they can be suppressed by parasitoid wasps that use them as hosts for their developing offspring. These wasps occur naturally in the environment and can be utilized in the control of these pests. We investigated how the number and species of pest aphids within the grain crops varied over the season, how their associated parasitoid wasp species and numbers varied over time, and how these organisms interacted at crop edges. In our study, aphid numbers remained low early in the season, and increased as the crop growth progressed. Wheat field edges can act as reservoirs for the aphids and wasps; however, this was not the case for canola field edges, likely due to the different plant hosts available. One wasp dominated this study throughout the canola fields, although it was only found in low numbers at field edges and in wheat fields. Within these areas, another wasp dominated. These findings can assist in the management of grain aphid pests.

**Abstract:**

In grain crops, aphids are important pests, but they can be suppressed by hymenopteran parasitoids. A challenge in incorporating parasitoids into Integrated Pest Management (IPM) programs, however, is that parasitoid numbers can be low during periods within the season when aphids are most damaging. Understanding the population dynamics of key aphid species and their parasitoids is central to ameliorating this problem. To examine the composition and seasonal trends of both aphid and parasitoid populations in south-eastern Australia, samples were taken throughout the winter growing seasons of 2017 and 2018 in 28 fields of wheat and canola. *Myzus persicae* (Sulzer) was the most abundant aphid species, particularly within canola crops. Across all fields, aphid populations remained relatively low during the early stages of crop growth and increased as the season progressed. Seasonal patterns were consistent across sites, due to climate, crop growth stage, and interactions between these factors. For canola, field edges did not appear to act as reservoirs for either aphids or parasitoids, as there was little overlap in the community composition of either, but for wheat there was much similarity. This is likely due to the presence of similar host plants within field edges and the neighbouring crop, enabling the same aphid species to persist within both areas. *Diaeretiella rapae* (M’Intosh) was the most common parasitoid across our study, particularly in canola, yet was present only in low abundance at field edges. The most common parasitoid in wheat fields was *Aphidius matricariae* (Haliday), with field edges likely acting as a reservoir for this species. Secondary parasitoid numbers were consistently low across our study. Differences in parasitoid species composition are discussed in relation to crop type, inter-field variation, and aphid host. The results highlight potential focal management areas and parasitoids that could help control aphid pests within grain crops.

## 1. Introduction

Grain production in Australia represents almost one-quarter of all agricultural exports and covers an area of 22 million hectares [1], with wheat constituting 55% of total arable crops sown [2]. Grain crops are usually grown in the Mediterranean and temperate climates of southern Australia, along the ‘grain belt’, with regions typically characterised by winter-dominant rainfall [3]. Annual rainfall of <600 mm is common, with half of Australia receiving <300 mm per year [4]. Wheat (*Triticum aestivum* L.) is grown in rotation with other cereals, particularly barley, and break crops such as pulses, canola (*Brassica napus* L.), and pastures [5]. Break crops can, in isolation, be profitable and increase diversity of income, in addition to improving grain yield [6]. ‘Canola’ is the trademark name provided by the Canadian Canola Association, referring to oilseed rape cultivars that produce oils with less than 2% erucic acid and meals with less than 30 μmol per gram glucosinolates in the oil-free meal [7]. *Brassica napus* was initially trialled in Australia during the early 1960s and commercial production began in 1969 using varieties imported from Canada [8]. Similar to Canada, conditions for crop growth in Australia can be harsh, impacting pest outbreak risk and causing food webs to be simpler than in regions with more favourable climates [9].

Aphids have become important crop pests across south-eastern Australia [10], and new pest aphids are arriving in Australian grain systems. The Russian wheat aphid, *Diuraphis noxia* (Mordvilko ex Kurdjumov), was discovered in Australia in 2016 and the potential economic impact associated with this pest remains unclear [11,12]. The introduction of a new aphid can alter community-wide interactions between aphids and their natural enemies. The invertebrate natural enemies of grain aphids range from many species of stenophagous and polyphagous predators to hymenopteran parasitoids, as well as fungal pathogens [13,14]. However, their relative impact has been disputed [15,16,17] due to a number of reasons. Firstly, the interactions between pests and natural enemy species that occur in crop environments will be greatly restricted when compared to all possible species interactions. Secondly, pest aphids must occur at the same time and place as suitable natural enemies, and at high enough densities to impact pest populations. Whilst the existence of high numbers of natural enemies within vegetation neighbouring a crop may be important, the greatest benefit is realised when individuals are able to move into the crop field to attack the pest [18]. Thirdly, benefits from natural enemies depend of their presence at susceptible stages of crop growth and arrival of pests in a field [19,20].

At present, Australian farmers rely heavily on broad-spectrum pesticide applications, most commonly pyrethroids, organophosphates and neonicotinoids, either applied as foliar applications or as seed dressings, to control grain aphids [21]. Due to the risk of aphids evolving resistance from the indiscriminate use of these chemicals [22], and the possibility of side-effects of pesticides on beneficial organisms [13], there is increasing interest in the implementation of Integrated Pest Management (IPM) strategies, including, but not limited to, biological control. Despite this, [23] noted that within Australian grains, interactions between pests and their natural enemies are not well understood. An understanding of natural enemies associated with pest species is essential for the application of biological control and IPM [24,25,26,27,28].

In this study, attention is primarily focused on the community of hymenopteran parasitoids and hyperparasitoids of grain aphids in south-eastern Australia. Waterhouse and Sands [29] list nine Australian grain aphid species (five that are pests of cereals and oilseeds) that are attacked by 13 primary parasitoids (nine in cereals and oilseeds) and ten secondary parasitoids. Additionally, these authors list nine primary parasitoids capable of parasitizing *D. noxia* in Australia, based on international literature [29]. This list, however, was compiled prior to the arrival of *D. noxia*, and not all of these species’ interactions will occur in grain fields each season. In this study, we used spatial and temporal surveys, and host rearing, to describe the diversity, abundance and activity of grain aphids and their natural enemies, including primary and secondary hymenopteran parasitoids, within Australian grain production landscapes. These landscapes are characterised by large field sizes interspersed with small, linear patches of non-crop vegetation such as trees used as shelterbelts, and field edges with introduced and native grasses and weeds. In south-eastern Australia, sowing of grain crops mostly occurs in autumn, with harvest in spring and early summer, followed by a dry fallow period over summer. To determine which parasitoid species are most likely to have an impact on pest aphid populations and provide pest management services for farmers, we address three questions:

- What are the temporal patterns in the grain aphid community (both abundance and species diversity)?
- What are the temporal patterns in the parasitoid and hyperparasitoid community (both abundance and species diversity)?
- What are the fine-scale spatial patterns in aphid–parasitoid interactions across the crop, field-boundary edge?

## 2. Materials and Methods

Between 2017 and 2018, we undertook extensive temporal sampling of invertebrates from a number of grain crops in the state of Victoria, Australia. On each sampling date, the growth stage of the crop, using Zadok’s growth chart for cereals [30] and an oilseed rape-specific chart for canola [31], was recorded. Ambient temperature at the time of sampling was noted. Photographs were also taken to catalogue differences in crop characteristics between the different fields and between sampling dates.

### 2.1. The 2017 Field Surveys

**Figure 1:**
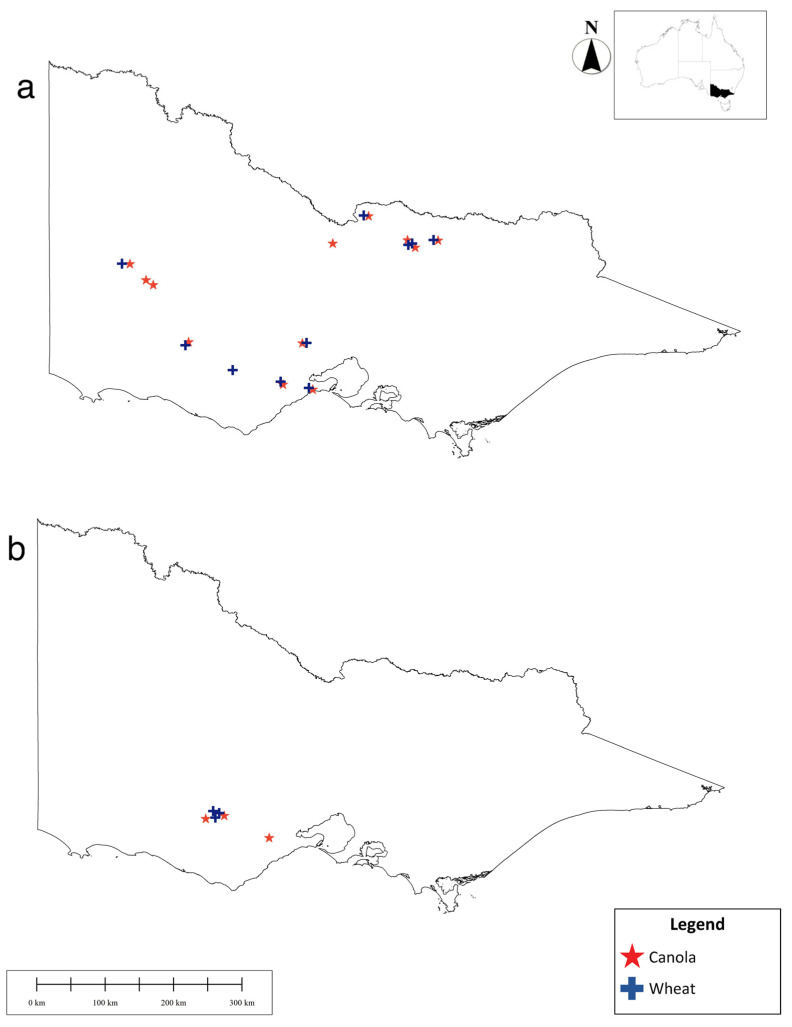
Location of (**a**) 2017 and (**b**) 2018 field sites, sorted into crop types (inset map depicts the state of Victoria at a national level) [32,33]. Map created in GlobalMapper v19.1.

In total, crop fields were surveyed 84 times over the 2017 growing season between 8 May (prior to sowing at each site) and 12 January 2018 (before harvesting/windrowing). Sixteen fields (10 of canola and six of wheat) were sampled every 6–12 weeks (either three or six times during the season), with a further six (two canola, four wheat fields) sampled twice throughout the season (due to being added at a later date) (Figure 1). Samples of aphids and natural enemies were obtained through yellow pan traps and direct searches. In the case of direct searching, we targeted field edges, crop plants that were stressed (e.g., due to waterlogging, soil compaction), and locations within each field known to be infested with aphids. At each site, crop plants were directly sampled once for aphids (alates, apterae and mummies) and aphid primary and secondary parasitoids for a total of two minutes, with the abundance and species of aphids recorded. All directly sampled aphids were placed within a resealable zip bag (S. C. Johnson & Son, Racine, WI, USA) along with host plant matter (keeping mummies and seemingly unparasitized aphids—those not engorged and golden in appearance—separate) for transport back to the laboratory.

On each sampling date, we also placed two yellow pan traps (filled with 150 mL of water and a drop of detergent until 31 July 2017, and subsequently 20% propylene glycol until the end of the season) on the ground at approximately 10 m and 30 m from the field edge. The change in solution was due to issues associated with freezing of the water solution during winter months. The pan traps were left in the field for 24 h and then decanted into a bottle for transport back to the laboratory.

We also sampled the surrounding vegetation of each crop field. All field edges were grassy refuges, consisting of a mixed composition of plant species, yet primarily Poaceae. Within each grassy edge, we randomly placed a single yellow pan trap on the ground and left it in the field for 24 h. As above, we then decanted the contents into a bottle for transport back to the laboratory.

### 2.2. The 2018 Field Surveys

In 2018, we repeated the temporal surveying of crop fields, again only focussing on wheat and canola. Six fields were sampled ten times between 18 May 2018 (prior to sowing at each site) and 23 November 2018 (before harvesting/windrowing) (Figure 1). All fields in 2018 were bordered by at least one boundary consisting of a shelterbelt of eucalyptus trees, with varying undergrowth, in addition to a second grassy edge. This allowed us to explore whether edge type influenced invertebrate abundance or species composition. Each field was sampled non-randomly every three weeks by direct searching and every six weeks by vacuum sampling.

**Figure 2:**
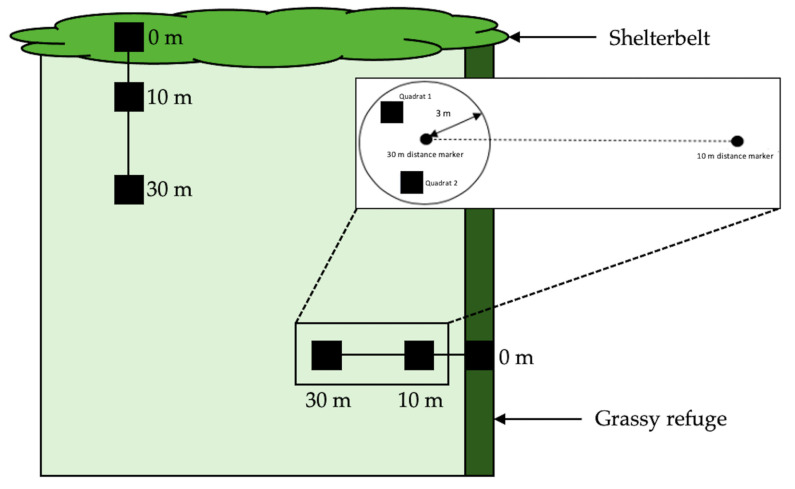
The arrangement of collection points at a field (NB, not to scale). Inset: Experimental set up for direct searching (quadrat 1) and vacuum sampling (quadrat 2), as undertaken in 2018.

Vacuum sampling was used as an alternative method to yellow pan trapping to sample invertebrates. Both techniques are suitable for aphid and parasitoid sampling [34,35,36,37]. Sampling occurred along two transects (one extending from the shelterbelt into the field, and one extending from the grassy edge into the field) (Figure 2). In 2018, at each sampling point, a quadrat (1 m × 1 m) was placed within a 3 metre radius at each distance along the transect. Invertebrates were collected within the quadrat by suction using a Stihl SH55 blower (STIHL Pty Ltd., Knoxfield, Australia) vacuum with a fine gauze mesh placed over the end of the vacuum tube for one minute. Once collected, samples were emptied into respective tubes of 80% ethanol in order to be transported to the laboratory.

In addition, we undertook direct searching for aphids (alates, apterae and mummies) and aphid parasitoids along each transect (Figure 2). This was undertaken by searching plants by hand for two minutes. As in 2017, all specimens collected were placed within a resealable zip bag (S. C. Johnson & Son, Racine, WI, USA) along with host plant matter (keeping mummies and seemingly unparasitized aphids—again, those not engorged and golden in appearance—separate for transport back to the laboratory).

### 2.3. Parasitoid Rearing and Invertebrate Identification

Once in the laboratory, directly caught aphids and aphid mummies were transferred to Petri dishes to observe parasitoid emergence. Petri dishes were made up with a 1% agar solution, within which 2–3 cotyledons of sprouting radish (*Raphanus raphanistrum* subsp. *sativus* L.) were inserted, and aphids were placed. Mummies were kept in separate Petri dishes in order to avoid subsequent parasitism of the aphids. Each dish was maintained at 20 °C (±2 °C), ~40% R.H. and a 16 Light (L):8 Dark (D) photoperiod for a period of three weeks. Leaves were changed weekly, or more regularly if they began to show signs of discolouration, if fungus growth was evident, or if the filter paper became too moist. Once reared, all parasitoids were stored in individual Eppendorf tubes with 80% ethanol and stored at 4 °C.

All aphidiines sampled directly from the field and those reared in the laboratory were sexed and identified morphologically to the species level (using keys produced by Rakhshani et al. [38] and Rakhshani et al. [39]) and/or genetically by DNA barcoding. These keys were selected from a variety of aphidiine keys available from different geographic areas, due to their level of detail, clear illustrations, and availability from 2017, ensuring the same keys were used throughout the sampling period. All listed traits were used to identify species. Parasitoids were selected for further genetic identification when their morphology varied from any identifying features noted within the keys. Furthermore, due to the variation in antennal segments between sexes, several males were barcoded, along with known females of particular species, to corroborate taxonomy. Secondary parasitoids were not identified to the species level due to resource limitations and because our main interest was focused on how they interacted with primary parasitoids.

All aphids and parasitoids collected from the yellow pan traps and vacuum samples were identified morphologically to the lowest taxonomic level possible and counted. Aphids were categorised into ‘apterae’ (unwinged), ‘alates’ (winged) and ‘mummies’ (parasitized; either winged or unwinged). Directly sampled aphids were identified morphologically to the lowest taxonomic level possible and counted. Blackman and Eastop [40] was the principal resource used to identify aphid species.

### 2.4. DNA Extraction and Barcoding of Parasitoids

DNA was extracted using a modified Chelex^®^ extraction method, adapted from [41], as detailed in Carew et al. [42]. One method used was the non-destructive DNA extraction from whole specimens. An individual parasitoid was placed within a 0.5 mL micro-centrifuge tube, along with 3 µL of Proteinase K (Roche Diagnostics Australia Pty. Ltd., Castle Hill, Australia) (20 mg/mL) and 70 µL of 5% Chelex^®^ solution (Bio-Rad Laboratories, Hercules, CA, USA), before being incubated in a 56 °C water bath for 60 min. Afterwards, the tube was transferred to a 90 °C water bath for 10 min. An alternative method involved removing a single hind leg from each parasitoid, before placing in a 1.7 mL sterile centrifuge tube along with two 3 mm glass beads, 3 µL of Proteinase K (20 mg/mL) and 70 µL of 5% Chelex^®^ solution. Samples were homogenised in a TissueLyser (Qiagen, Hilden, Germany) for 2 min at 25 Hz, and after centrifuging, were incubated in a 65 °C water bath for 60 min, before transferral to a 90 °C water bath for 10 min. Prior to PCR, tubes were spun at 13,000–14,000 rpm for five minutes in a D3024 high-speed microcentrifuge (DLAB Scientific, Beijing, China), and aqueous DNA was pipetted from just above the Chelex^®^ resin (Bio-Rad Laboratories, Hercules, CA, USA), ensuring the resin remained in the tube.

For each sample, we targeted a 658 base pair fragment of the cytochrome *c* oxidase subunit 1 gene (*co1*). PCRs were undertaken using a 1/10 dilution of the DNA extractions and amplifying the samples with the “universal” invertebrate primer pair LCO1490/HCO2198 [43]. Reactions contained a final concentration of 1x Standard Taq Reaction Buffer (New England Biolabs, Ipswich, MA, USA), 2.5 mM MgCl_2_, 0.5 µM each primer, 0.2 mM dNTPs, 2.4 U IMMOLASE DNA Polymerase (Bioline, London, UK) and 3 µL diluted DNA, in a reaction volume of 30 µL. Amplicons were sent to the Australian Genome Research Facility (AGRF) for sequencing, before forward and reverse sequences were assembled and trimmed using Geneious version 9.1.8 (https://www.geneious.com). Sequences were searched against the Genbank database (http://www.ncbi.nlm.nih.gov) and Barcode of Life Data System database (BOLD; http://www.barcodinglife.org [44]) to ascertain their identity.

### 2.5. Data Analysis

For analyses, 2017 data were used for comparative purposes only, due to the low numbers of repeat sampling. The relative frequency of apterae, alates, and mummies was calculated as a percentage of the total aphids sampled (collections of ten or less aphids were omitted). Generalized linear models (GLM) were used to test whether the sampling method, field, or location (in-field versus shelterbelt versus grassy refuge) had an effect on the response variables (counts of aphids, parasitoids or mummies, log(x + 1) transformed where required for normality, and proportions of alates). Data were summed across each sampling point within a field and a mean count was taken for these analyses. Two-way interactions between the aforementioned factors were also investigated via GLMs.

Pairwise comparisons were used to determine differences between the logged (log(x + 1)) mean abundance of alates and apterae directly sampled and vacuum sampled per point, across all fields, in 2018.

In 2018, the effects of inter-field variation, field versus edge effects, and crop growth stage on aphid and parasitoid composition were analysed using multiple response permutation procedures (MRPP), with Euclidean distance as a similarity measure. Data were summed across sampling points within a field, with shelterbelt and grassy refuge kept separate. Data collected before trip 7 for direct sampling and rearing in 2018, and trip 6 for vacuum sampling in 2018, were excluded due to the high proportion of zero data. For crop growth stage effects, data were kept for each time point, yet for the other factors, data were summed across all time points.

All analyses were conducted using Minitab version 19.1.0.0 [45], with the exception of the multiple response permutation procedures (MRPPs), which were performed in R version 4.0.1 [46], using RStudio version 1.3.959 [47]. Significant results are displayed in bold.

## 3. Results

### 3.1. Seasonal Abundance and Community Composition of Crop Aphids

**Figure 3:**
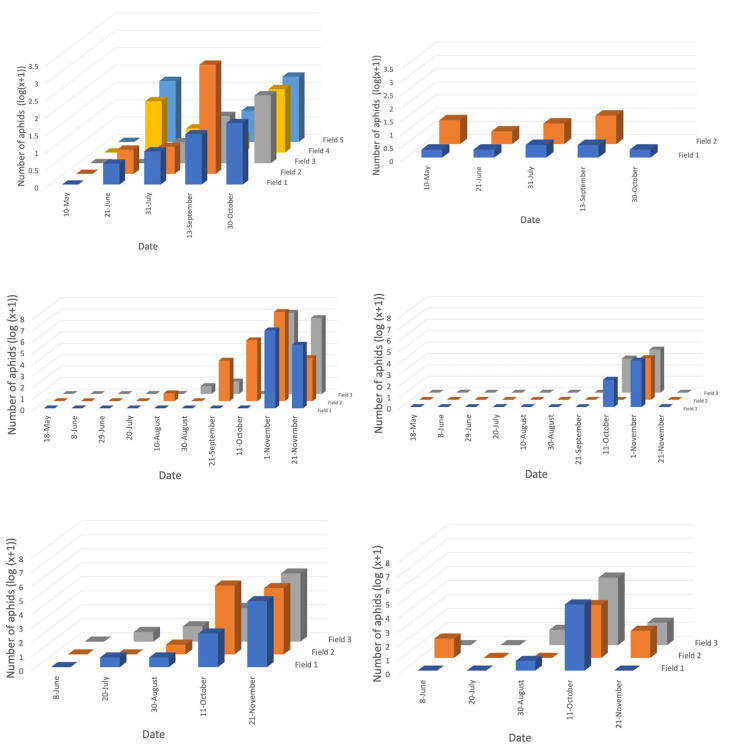
Logged numbers of all aphids per field retrieved by direct searching for two minutes within (**a**) canola and (**b**) wheat fields in 2017, direct searching for two minutes within (**c**) canola and (**d**) wheat fields in 2018, and vacuum sampling for one minute within (**e**) canola and (**f**) wheat fields, in 2018. Bar colours differentiate fields. Fields surveyed less than twice were omitted.

For both years, crop growth stage significantly affected the abundance of directly collected aphids in canola, as well as those vacuum sampled in 2018, with numbers increasing as growth stage progressed (Figure 3a,c,e). For wheat fields, crop growth stage also affected the number of aphids directly sampled in 2018 but did not for those directly sampled in 2017 or vacuum sampled in 2018 (Figure 3b,d,f). Canola yielded five times as many aphids as wheat, for both collection methods in 2018. Furthermore, three times as many aphids were sampled directly than by vacuuming overall in 2018.

**Figure 4:**
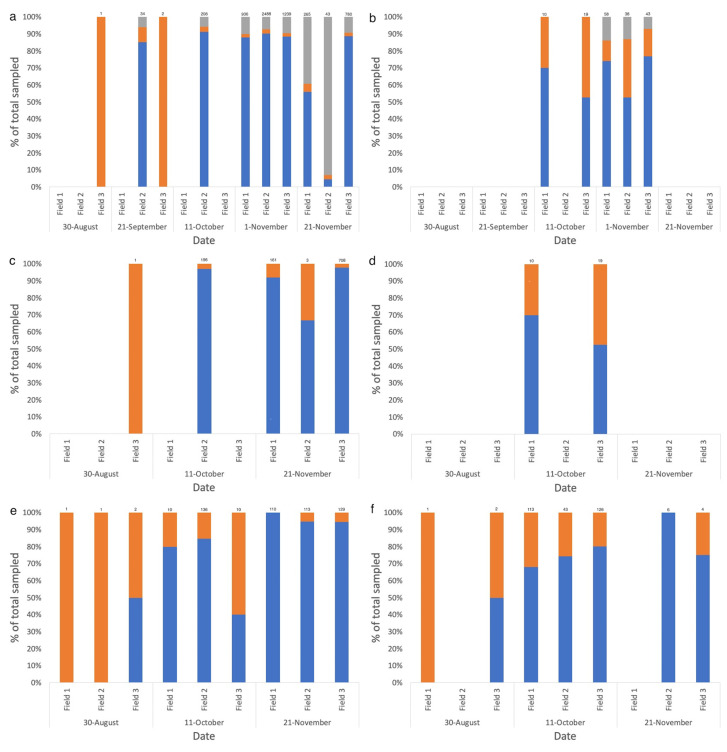
Relative frequency of apterae and alates from the total number of aphids (**a**) directly sampled within canola fields, including mummies, (**b**) directly sampled within wheat fields, including mummies, (**c**) directly sampled within canola fields, excluding mummies, (**d**) directly sampled within wheat fields, excluding mummies, (**e**) vacuum sampled within canola fields, and (**f**) vacuum sampled within wheat fields, in 2018.

Pairwise comparisons determined a significant difference between alate and aptera collections from direct sampling, with apterae collected in greater numbers (mean = 0.553, standard deviation (SD) = 0.128) than alates (mean = 0.121, SD = 0.027; t(35) = −4.13, *p* = **0.001**). This was also true of those aphids vacuum sampled in 2018, with apterae collected in higher numbers (mean = 0.855, SD = 0.128) than alates (mean = 0.357, SD = 0.238; t(35) = −4.26, *p* = **0.001**). The proportion of alates sampled directly and via vacuum from canola or wheat fields did not change with crop growth stage. The relative frequency of apterae, alates, and mummies was calculated as a percentage of the total aphids sampled. The relative frequency of alates for both sampling methods was higher earlier in the season for canola yet varied for wheat (Figure 4). In canola, mummification rate significantly increased from 3–4% in September/October, increasing as crop growth stage progressed and peaking at 20% by the end of November (Figure 4). This was not the case in wheat fields, with crop growth stage having no effect on the proportion of mummies sampled.

**Figure 5:**
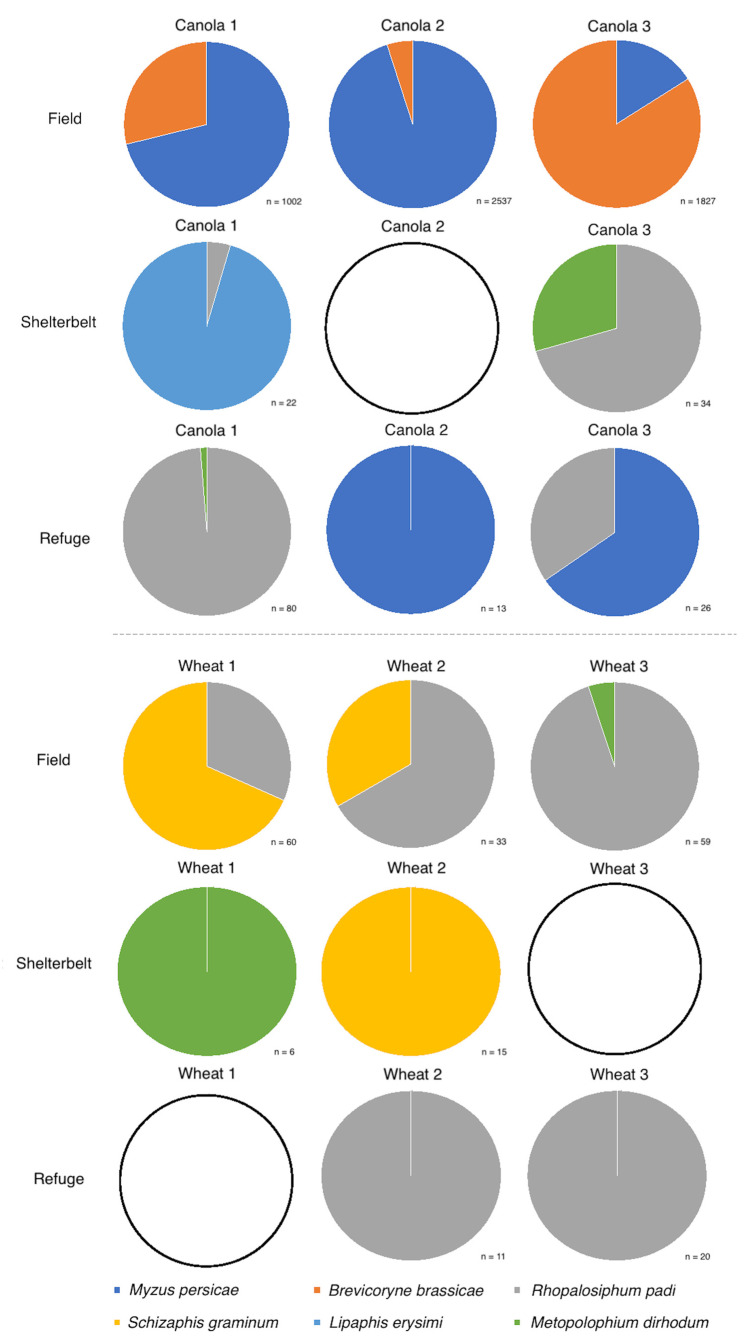
Aphid species composition from two-minute direct sampling undertaken in canola and wheat crops in 2018. n = number of aphids sampled. Where n < 5, pie charts are shown as empty circles.

In 2017, crop growth stage did not have a significant effect on the directly sampled aphid species composition, for either canola (MRPP, Chance-corrected estimate of the proportion of the distances explained by group identity (A) = 0.889, *p* = 0.176) or wheat (MRPP, A = −0.140, *p* = 0.621). In 2018, for the direct samples, the green peach aphid (*Myzus persicae* (Sulzer)) dominated the aphid populations in two canola fields, while the cabbage aphid (*Brevicoryne brassicae* L.) dominated in one (Figure 5). Within wheat, the oat aphid (*Rhopalosiphum padi* (L.)) dominated in two fields, with the greenbug (*Schizaphis graminum* (Rondani)) dominating in one (Figure 5). The effect of inter-field variation was not found to be significant on directly sampled aphid species composition for either canola (*p* > 0.05) or wheat (*p* > 0.05), likely due to the limited number of species sampled for both crop types. Crop growth stage had a significant effect on the directly sampled aphid species composition, for canola (MRPP, A = 0.442, *p* = **0.001**) and wheat (MRPP, A = 0.176, *p* = **0.001**) in 2018 across every sampling trip.

### 3.2. Seasonal Abundance and Community Composition of Parasitoids

A summary of parasitoids reared from directly sampled mummies in 2017 and 2018 is provided in Table 1. Parasitoids were categorised into primary parasitoids and secondary parasitoids (hyperparasitoids and mummy parasitoids; defined as those attacking the living aphid with delayed development and those attacking the mummified aphid with instantaneous development, respectively [48]).

**Figure 6:**
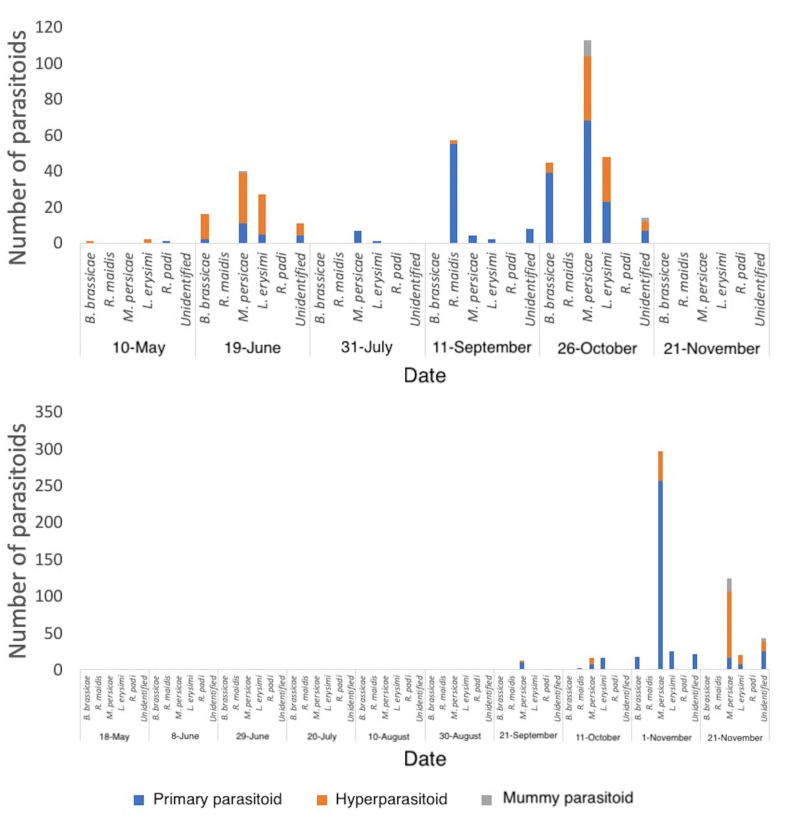
Number of mummies collected within and surrounding all fields in (**a**) 2017 and (**b**) 2018, within two minutes, along with the relative frequency at which primary parasitoids, hyperparasitoids, and mummy parasitoids emerged.

In both years, *Diaeretiella rapae* (M’Intosh) was the most commonly reared primary parasitoid for the majority of aphid species. Parasitoids were reared from canola fields (and surrounding areas) earlier in the year than those from wheat fields (and surrounding areas). Abundance of aphid parasitoids directly sampled in canola, and vacuum sampled in wheat, varied across crop growth stage, with numbers increasing as crop growth stage progressed (Figure 6).

In 2018, within canola fields, primary parasitoid numbers greatly increased from October until the end of the season, regardless of sampling method (Figure S1). Primary parasitoids were found in very low abundance and no secondary parasitoids were directly collected in wheat fields in 2018 (Figure S1). Parasitoids were reared later in the season in 2018 than in 2017 (Figure 6). Hyperparasitoids were reared in greater numbers earlier in the growing season of 2017, reaching 76% in June, peaking again in October. In 2018, hyperparasitoids peaked in late November, reaching 62% (Figure 6). No secondary parasitoids were reared from either crop edge or within wheat fields in 2018. Mummy parasitoids were only reared from aphids collected within canola fields at the very end of the season, for both years. For both years combined, parasitism rates were estimated as 41% in canola, and 17% in wheat.

**Figure 7:**
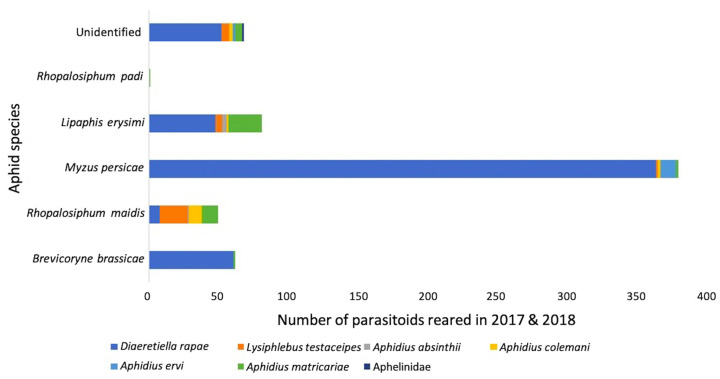
Primary parasitoid species composition of aphid species from fields and surrounding vegetation in 2017 and 2018, reared from mummies collected directly sampled for two minutes.

*Diaeretiella rapae* comprised 60% of all parasitoids reared, and 89% of all primary parasitoids reared in 2018 (Table 1). Furthermore, *D. rapae* predominated parasitism for each aphid species, constituting 95% of all parasitoids reared from *B. brassicae*, 63% from *M. persicae*, 29% from *L. erysimi. Rhopalosiphum maidis* (Fitch) was the exception to this rule, with no *D. rapae* reared from this aphid in 2018 (Table 1). Only two parasitoids were reared from this species: *Aphidius matricariae* (Haliday), and *A. absinthii* Marshall. In 2017, *D. rapae* constituted 65% of all parasitoids reared from *B. brassicae*, 48% of *M. persicae,* 38% of *L. erysimi*, 16% of *R. maidis,* in addition to 44% of all parasitoids reared, and 73% of all primary parasitoids reared (Table 1). Once again, no *D. rapae* were reared from *R. padi*. *Lysiphlebus testaceipes* dominated *R. maidis* parasitism, and a single *A. matricariae* was reared from *R. padi* (Table 1). The general trends for both years combined are shown in Figure 7, with *D. rapae* constituting 78% of total parasitoids.

In 2018, direct and vacuum sampling showed a steady increase in numbers of *D. rapae* within canola and wheat fields, yet the reared *D. rapae* peaked in early November, decreasing in late November, suggesting a decrease in reproductive rate for this species (Figures S2–S4). Direct samples from within canola fields showed a similar composition of parasitoid species throughout the growing season, with *D. rapae* constituting 100% of parasitoids sampled in September and October, 89% in early November, and 90% in late November 2018 (Figure S2). Mummy parasitoids were directly sampled only in canola in late November, and hyperparasitoids in early and late November (Figure S2). Furthermore, hyperparasitoids dominated *L. erysimi* parasitism. Only one parasitoid was directly sampled from the edge of a canola field, which was *A. matricariae* in early November. Furthermore, only one parasitoid was directly sampled from the edge of a wheat field, which was *L. testaceipes,* in early November (Figure S2).

### 3.3. Fine-Scale Spatial Patterns in Aphid–Parasitoid Interactions across Field Edges

**Figure 8:**
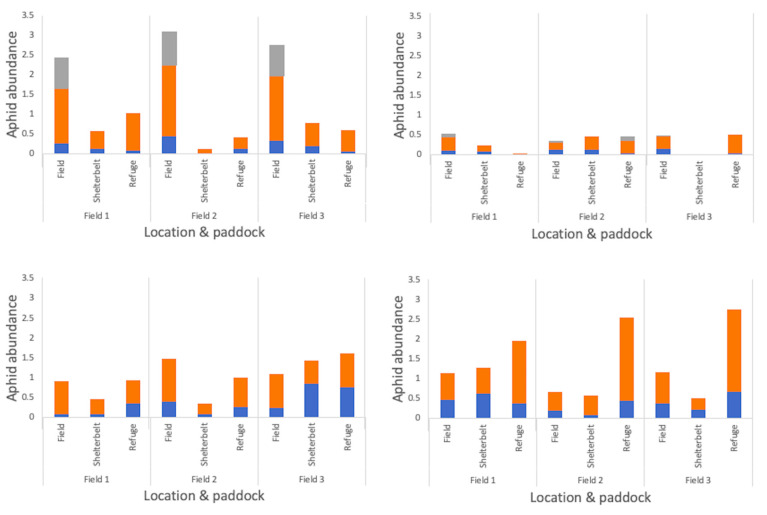
Logged numbers of aphid forms depending on location within and surrounding each crop in 2018 for those directly sampled for two minutes in (**a**) canola, and (**b**) wheat, and vacuum sampled for one minute in (**c**) canola, and (**d**) wheat.

Aphid population trends in 2018 were the same for both edge types (‘grassy refuge’ and ‘shelterbelt’), peaking in October in the vacuum samples and in early November, when directly sampled. There was an increase in the number of aphids directly sampled within crop fields in early November, but not at the edge of the field, where there was a decline in the number of aphids sampled from their population peak in October (Figure S5). Within and surrounding canola fields, the location (in-field versus shelterbelt versus grassy refuge) was found to affect the number of aphids collected, with greater numbers directly sampled within the field than at either edge type in 2018, which was also the case in 2017 (Figure 8 and Table 2 and Table 3). In 2017, greater proportions of alates were sampled in the grassy refuge compared with alates collected in field (Table 2). In 2018, alate proportions were not affected by location within and surrounding canola fields, but were for wheat fields, with lower proportions collected at the grassy refuge in comparison to the shelterbelt and field (Figure 8 and Table 3). Significantly higher numbers of mummies were collected within the canola field than at either edge type, although this was not the case for wheat fields (Figure 8 and Table 3).

There was little overlap in aphid species composition between some fields and their bordering vegetation. This was particularly the case for canola, where there was a significant difference in aphid species composition within a field compared with the edge (MRPP, A = 0.019, *p* = **0.006**), unlike for wheat (MRPP, A = −0.003, *p* = 0.751) (Figure 5). Although *M. persicae* peaked within canola fields in November, this species spiked in numbers at the field edge prior to any other aphid species, in September, staying consistently high the following month (Figure S6). In wheat fields and their surrounding vegetation, however, half the number of aphid species (*R. padi*, *S. graminum*, and *M. dirhodum*) were directly sampled compared with their canola counterparts, with aphid populations peaking in early November, both within and at the edge of the fields, and decreasing by the next visit, except for *R. padi* populations which increased in early October (Figure S6).

**Figure 9:**
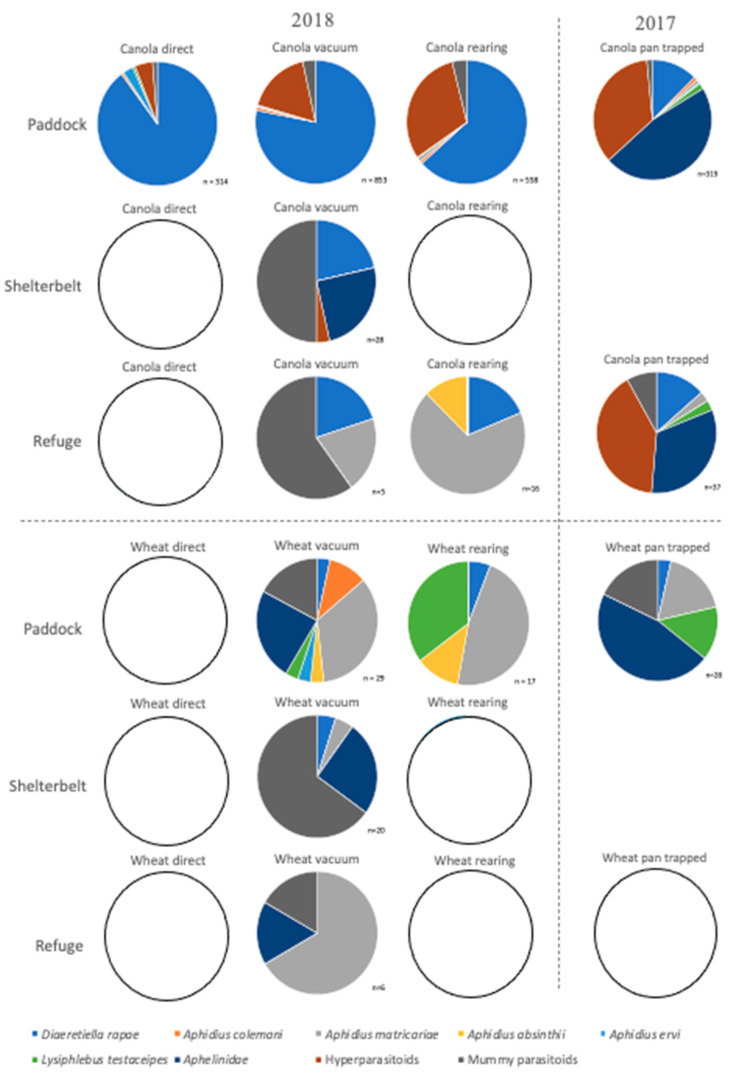
Parasitoid species sampled within fields and their neighbouring vegetation, sampled directly for two minutes, by vacuum for one minute, through rearing of mummies from directly sampled mummies over two minutes, or from pan trapping over 24 h, in 2017 and 2018. Where n < 5, pie charts are shown as empty circles.

Location significantly affected the number of parasitoids collected within and surrounding canola fields, with more individuals sampled within the canola fields than at either edge type in 2018, as was the case in 2017, with higher numbers collected in the field compared with the grassy refuge (Figure 9 and Table 2 and Table 3). Furthermore, sampling method affected the number of parasitoids collected within and surrounding both crop types, with vacuum sampling producing the greatest numbers, followed by rearing, and then direct searching, for both crop types in 2018 (Figure 9 and Table 2).

*Diaeretiella rapae* greatly increased in abundance at the end of the season within canola fields, reaching 78%. Within fields, only mummy parasitoids were sampled early in the season (early June), and no parasitoids were sampled in late July or late August for either crop type, both within, and at the edge, with the exception of one *Aphelinus* sp. (primary parasitoid) at the edge of a canola field. *Diaeretiella rapae* and hyperparasitoids were the only parasitoid types reared from within canola fields until early November. *Diaeretiella rapae* was also the only species reared from the edge of canola fields in late September, before *A. matricariae* became the most commonly reared parasitoid in October, with no parasitoids reared after that month (Figure S4). Like the canola edge species composition, the vegetation neighbouring wheat fields yielded mostly *A. matricariae* from October. Within the wheat fields, only *L. testaceipes* was reared in October, with *A. matricariae* predominating in early November. No parasitoids were reared within or surrounding wheat fields from early November (Figure S4).

Inter-field variation had no effect on the species composition of aphid parasitoids for either crop, for any sampling method, apart from vacuum-sampled parasitoids in and surrounding canola crops (Table 4). The species composition of parasitoids did not vary between the crop edge and wheat field for any sampling method. However, it varied between canola fields and surrounding vegetation for every sampling method used, with a greater proportion of *D. rapae* sampled within canola fields than within the neighbouring vegetation (Figure 9 and Table 4). There was also variation between the refuge and field for pan trapped parasitoids in canola in 2017, with a greater diversity of parasitoids sampled within the field (Figure 9 and Table 4). Across crop growth stage, aphid parasitoid species composition did not vary for either crop type, for any sampling method, apart from in the case of those aphid parasitoids vacuum sampled within wheat fields (Table 4). Within canola fields, *D. rapae* was always directly sampled and vacuumed in greater proportions than when it was reared, yet greater proportions of hyperparasitoids were reared than were sampled (Figure 9). Sampling of parasitoids within wheat crops was not a good indication of those species reared, with a greater proportion of *D. rapae* vacuumed than reared (Figure 9).

## 4. Discussion

Waterhouse and Sands [29] listed five aphid species within Australian cereals and oilseeds, with their associated parasitic hymenoptera totalling nine primary parasitoids and ten secondary parasitoids. The same five aphid species were recorded during this study, with the addition of *S. graminum*. Whilst *D. noxia* has recently arrived in Australia, with this species first identified in this country in 2016, it was not recorded at any field sites in this study. Regarding primary parasitoids recorded by Waterhouse and Sands [29], six of the nine listed were sampled here, in addition to *A. matricariae*. Secondary parasitoids were not identified to the species level during this study. However, the hyperparasitoid genera *Alloxysta* Foerster and *Phaenoglyphis* Förster, both Figitidae, were sampled. Furthermore, of those listed by Waterhouse and Sands [29], the mummy parasitoid genera *Dendrocerus* Ratzeburg and *Pachyneuron* Walker were sampled, but *Syrphophagus* sp. Ashmead, *Euryischomyia flavithorax* Girault and Dodd, and *Moranila comperei* (Ashmead) were not. Of those not listed, *Asaphes* sp. Walker and *Coruna* sp. Walker were also recorded. Within this study, parasitism rates were estimated as 41% in canola, and 17% in wheat in the period 2017–2018. This demonstrates that whilst there is a core group of species interactions in the grain aphid–parasitoid community, there is some variability across the short growing seasons and locations as to the realised species interactions.

### 4.1. What Are the Temporal Patterns in the Grain Aphid Community?

Seasonal weather and crop growth stage, in addition to the interaction between the two, appear to be the most important factors influencing the temporal patterns of grain aphids in our study. Climatological conditions, such as temperature, can govern species distribution and abundance, community composition, and ecosystem function [49,50]. The lack of spatial variation in aphid community composition between fields, with the absence of ‘field’ as a significant factor, supports this hypothesis for both crop types. Seasonal factors appear to override any spatial variation in community composition expected with sampling from geographically dispersed fields, with crops growing at similar rates and sown at a similar time of year.

In 2018, although most aphid activity occurred from September/October until the end of the season, abundances of early season aphids were low. Early season aphids are of particular economic importance, due to their capacity to initiate population increases to potentially damaging levels and their ability to introduce viruses into crops at a vulnerable stage [51]. In 2017, early season aphids were sampled at greater numbers in canola than during the subsequent year. The time of year at which aphids colonize crops may be due to the crop growth stage, the presence of grassy edges and/or chemical effects [52,53,54]. High inter-annual variability in the trophic web has been recorded across two consecutive sampling years by Von Baaren et al. [9], with the authors suggesting that populations are likely limited by the (colder) weather before and during the growing season. In our study, crop growth stage significantly affected aphid species composition, for all trapping methods, within both crop types sampled in 2018. Aphid population growth is also known to be affected by weather (namely, temperature, rainfall, and wind) [55], with warmer temperatures and drier winters leading to a greater abundance of aphids over a canola growing season (Barton et al., in submission). Here, aphid collections were highest at the end of the growing seasons, closer to the warmer, summer months (with averages of 15–16 °C and highs of ~30 °C). This correlated with the peaking of parasitoids. Natural enemy increases are likely attributable to the subsequent decrease in aphid numbers at the end of the sampling period, particularly when acting in tandem [56]. Additionally, aphid population declines could be attributed to a reduction in plant quality after podding and during windrowing/harvest.

In 2018, direct searching yielded aphids later in the season than vacuum sampling, but more aphids were collected through the former method. To identify early season colonization of crops by aphids, vacuum sampling may be preferable. Both methods are active, though direct searching is more difficult to replicate, particularly as some aphid species, such as *D. noxia* inhabit areas deep within the leaf whorls of cereals [11,57]. Lower numbers of aphids were directly sampled from wheat than from canola, perhaps due to the broad leaves and prostrate growth structure of the latter, making it easier to sample. The effectiveness of pan trapping, a passive sampling method, is difficult to compare with other sampling techniques because colour and preservative can affect catches [58,59]. Generally, however, this trapping method is most effective at catching flying invertebrates [60].

### 4.2. What Are the Temporal Patterns in the Aphid Parasitoid Community?

*Diaeretiella rapae* was the most common parasitoid in both years and the most commonly reared parasitoid from aphids collected, with the exception of the *Rhopalosiphum* genus. The large numbers of *M. persicae* and *B. brassicae,* both brassica aphids, likely contributed to the abundance of this parasitoid, as it is known globally to prefer aphid hosts on cruciferous plants [61,62,63,64,65]. Furthermore, *D. rapae* has the ability to withstand the defensive kicking behaviour displayed by the most commonly sampled canola aphid, *M. persicae* [66].

Of the parasitoids directly sampled within canola fields, *D. rapae* constituted all parasitoids collected in September and October 2018, and the majority towards the end of the season. The preponderance of this species over other aphidiines may be due to its ability to thrive under cooler temperatures and lower humidities [67]. *Diaeretiella rapae* was found to be the most common parasitoid in canola in Oklahoma, USA [68], the predominant aphidiine during the establishment of aphid colonies in canola in Brazil [69], and the earliest parasitoid to mummify aphids in cabbage crops in the UK [70]. In our study, *D. rapae* was the only primary parasitoid reared from mummies within canola fields until early November, which could reflect a superior competitive ability and/or an ability to withstand insecticide applications [71,72].

Vacuum-sampled mummy parasitoids were detected at consistently high abundances throughout the growing season within vegetation neighbouring crop fields. Within these fields, mummy parasitoids were sampled in early June, yet no parasitoids were sampled in the subsequent two months, suggesting the field edges may act as parasitoid reservoirs. Furthermore, mummy parasitoids were only directly sampled and reared from mummies collected in canola in late November 2018, suggesting that they may not successfully reproduce within fields during the growing season. Conversely, hyperparasitoids were reared throughout the season.

Secondary parasitoid numbers increased at a slower rate than that of the primary parasitoids, slightly lagging behind as might be expected, given they require primary parasitism to reproduce [73]. Similarly, in a study surveying cereal aphids and their associated parasitoids in Denmark, hyperparasitism levels generally increased during the season, so late-developing primary parasitoids had a higher risk of themselves being parasitized [74]. No secondary parasitoids were collected or reared from wheat fields during the growing season, which could reflect low primary parasitism, meaning secondary parasitoids were unable to locate mummies for their own oviposition [75].

The proportion of mummies from total aphids, increasing from 1% in early November 2018 to 15% in late November 2018, suggests that the parasitism rate may increase with crop growth stage; a trend recorded in previous studies [76,77]. In canola fields in Brazil, mummy trends followed similar temporal patterns, but peaked during the crop elongation phase rather than prior to podding [69]. Conversely, in a study from Pakistan, the population density of aphids was directly proportional to that of mummies [78]. Of the canola fields sampled in our study in 2018, one was directed harvested; two others were windrowed—a method of cutting and leaving the grain to dry before being harvested. In the harvested crop, mummification continued to increase at the end of the season, rather than peaking on the penultimate visit, which occurred in windrowed crops. When crops are directly harvested, aphids and natural enemies will disperse onto other host plants [79]. Windrowed plants provide shelter and continuous food sources for pests and beneficials alike [80].

### 4.3. What Are the Fine-Scale Spatial Patterns in Aphid–Parasitoid Interactions?

Aphid–parasitoid interactions observed in the edges surrounding canola fields and within the fields were very different, whereas for wheat fields there were some similarities between the two. The factor ‘Location’ was found to affect aphid, mummy, and aphid parasitoid abundance at the canola sites, with greater numbers collected within canola fields than either edge type surrounding the fields.

Both edge types (shelterbelts and grassy refuges) can potentially be beneficial for invertebrates [81,82]. However, in this study, parasitoids were directly sampled only from grassy edges, reared in greater numbers from this edge type, and reared from within fields in greater numbers within the vicinity of grassy edges compared with shelterbelts. Insecticide-free grassy edges, either native or weedy [83], can host parasitoids, enabling them to move into the crop at distances of up to 120 m [84]. Flower-rich edges may provide greater nectar (and nutrition) than perennial crops [85], increasing adult parasitoid life span and egg production [86], and in turn increasing aphid parasitism [87]. *Diaeretiella rapae* is more strongly associated with cruciferous plants [88], which, in our study, were more common in the grassy edges than shelterbelts. *Diaeretiella rapae* was the first primary parasitoid reared from neighbouring vegetation. However, it was later exceeded by *A. matricariae*. Although *D. rapae* has a potential host range of approximately 60 aphid species, only five to six are commonly attacked [89], including many not recorded here. This suggests that *A. matricariae* may have had an advantage in using host aphids available in the non-cruciferous neighbouring vegetation.

Towards the end of the growing season, more aphids were sampled from canola fields than at the edge. Tall, dense crops provide greater feed and shelter, and the preceding flowering stage can attract aphids by the yellow colour of the flowers [90,91,92,93,94]. The difference in aphid species composition in canola fields compared with the edges could be explained by the different host plants at each location. Only one species of aphid, *M. persicae*, was present within both canola edge types. In the wheat fields, the same three aphid species were found in both wheat fields and edge locations. However, when separated into ‘shelterbelts’ and ‘grassy refuges’ neighbouring wheat fields, only *R. padi* was sampled; the same species was found within each wheat field. This is likely due to the grassy edges composing mostly of Poaceae plant species. Grasses are alternative host plants of cereal aphids [95], providing reproduction habitat for pests [96] and potentially allowing movement between wheat fields and neighbouring vegetation. Similar results may be expected for canola crops if cruciferous plants had been abundant within the crop edges, as suggested by Severtson et al. [97].

There was a greater diversity of aphid parasitoids sampled within the field in comparison to either type of neighbouring vegetation (for both crop types), perhaps due to the greater number and diversity of aphid hosts sampled. Parasitoid species composition differed across canola fields and edges, with a greater proportion of *D. rapae* and primary parasitoids sampled within the fields than within the neighbouring vegetation. In contrast, the dominant aphid parasitoid species (*A. matricariae*) found in wheat fields was also found in high proportions in neighbouring vegetation. Additionally, this species was found in greater numbers than *D. rapae* in the edges of canola fields. These results suggest that both edge types act as reservoirs for *A. matricariae*, in addition to mummy parasitoids, which is useful for enhancing parasitism within wheat fields. However, for canola fields, where *D. rapae* predominates, this species was rarely found in either edge type. Most likely, *D. rapae* uses cruciferous plants (such as volunteer canola and radish) to maintain populations, although further research is required.

Although parasitism rates were low in our study, parasitoids are still likely to be an integral part of a larger system of pest control in grain production landscapes. There is consistency across geographic area in the aphid parasitoid community for each crop type, with the temporal patterns appearing to be caused by seasonal weather and crop growth stage. Yet, the aphid parasitoid community, like the aphid community, is different between canola and wheat crops. This suggests that different methods of management are required to enhance natural populations of parasitoids in different crops. Increasing plant diversity and managing non-crop vegetation can be effective for enhancing conservation biological control in cropping systems [98,99].

## 5. Conclusions

This is the first comprehensive study of grain aphids and their associated parasitic fauna within Australia. Aphids and parasitoids generally increased in abundance as the cropping season progressed, with community compositions changing as crops reached later growth stages. Although secondary parasitoids were present throughout the year, either inactively within field edges, such as mummy parasitoids, or actively parasitizing aphids, such as hyperparasitoids, numbers remained low until the end of the growing season. Sampling techniques affected the abundance of pests and parasitoids detected, as well as the diversity of parasitoids collected, with vacuum sampling most likely to detect early crop colonization by pests. Additionally, inter-field variation, crop type and aphid species all affected parasitoid species composition. *Diaeretiella rapae* was the most predominant parasitoid within sampled fields, yet was found in low abundance in neighbouring vegetation, where *A. matricariae* was dominant. These species appear to be particularly important biological control agents at different stages of crop growth and in different crop types.

## Figures and Tables

**Table 1:** Summary of parasitoids reared from mummies directly sampled for two minutes in 2017 and 2018.

Hymenopteran parasitoids sampled	Aphid Host													
	*Brevicoryne brassicae*		*Rhopalosiphum maidis*		*Myzus persicae*		*Lipaphis erysimi*		*Rhopalosiphum padi*		Unidentified		Total	
	2017	2018	2017	2018	2017	2018	2017	2018	2017	2018	2017	2018	2017	2018
**Primary parasitoids: Hymenoptera: Braconidae**														
*Diaeretiella rapae*	41	20	8	0	78	286	29	19	0	0	18	34	174	359
*Lysiphlebus testaceipes*	0	0	20	0	0	1	0	5	0	0	5	1	25	7
*Aphidius absinthii*	0	0	0	1	0	0	0	3	0	0	0	0	0	4
*Aphidius colemani*	0	0	9	0	2	0	0	1	0	0	2	0	13	1
*Aphidius ervi*	0	0	0	0	9	2	0	0	0	0	0	2	9	4
*Aphidius matricariae*	0	1	11	1	1	1	1	23	1	0	1	4	15	30
Primary parasitoids: *Aphelinus* spp.	0	0	0	0	0	0	0	0	0	0	1	0	1	0
**Hyperparasitoids**														
*Alloxysta/ Phaenoglyphis* spp. (Hymenoptera: Figitidae)	22	0	2	0	65	145	47	13	0	0	12	13	148	171
**Mummy parasitoids**														
*Dendrocerus* spp. (Hymenoptera: Megaspilidae)	0	0	0	0	6	0	0	0	0	0	2	0	8	0
*Pachyneuron* spp. (Hymenoptera: Pteromalidae)	0	0	0	0	4	17	0	0	0	0	0	5	4	22
Total	63	21	50	2	161	435	77	64	1	0	41	54	397	598

**Table 2:** GLM results for aphid abundance, proportion of alates, and aphid parasitoid abundance across canola and wheat fields from 24 h yellow pan trapping undertaken during the 2017 season. Fields and locations (field versus refuge) are compared.

Organism and Crop Monitored	Factor	Mean Square (MS)	F_(df1, df2)_	*p*
Aphid abundance ^ in canola	Field	0.0778	4.98_(7, 15)_	0.025
	Location (field vs. refuge)	0.1989	12.72_(1, 15)_	0.009
Aphid abundance ^ in wheat	Field	0.1289	1.41_(7, 15)_	0.330
	Location (field vs. refuge)	0.1419	1.55_(1, 15)_	0.253
Proportion of alates in canola	Field	0.1924	0.52_(7, 15)_	0.796
	Location (field vs. refuge)	0.0014	0.00_(1, 15)_	0.953
Proportion of alates in wheat	Field	0.7217	7.32_(7, 15)_	0.009
	Location (field vs. refuge)	0.0084	0.09_(1, 15)_	0.778
Aphid parasitoid abundance^ in canola	Field	0.0950	4.64_(4, 9)_	0.083
	Location (field vs. refuge)	0.1893	9.25_(1, 9)_	0.038
Aphid parasitoid abundance ^ in wheat	Field	0.0324	2.30_(3, 7)_	0.256
	Location (field vs. refuge)	0.0001	0.01_(1, 7)_	0.932

^ Abundance logged using (log(x + 1)). Significant results are displayed in bold.

**Table 3:** GLM results for aphid abundance, proportion of alates, mummy abundance, and aphid parasitoid abundance across canola and wheat fields from two-minute direct sampling and one-minute vacuum sampling undertaken during the 2018 season. Sampling methods, fields and locations (field versus refuge), and interactions between these factors, are compared. Results of GLM for both crop types directly and vacuum sampled in 2018.

Organism and Crop Monitored	Factor	MS	F_(df1, df2)_	*p*
Aphid abundance ^ in canola	Sampling method	0.0400	1.13_(1, 17)_	0.348
	Field	0.0800	2.25_(2, 17)_	0.221
	Location (field vs. shelterbelt vs. refuge)	1.0133	28.53_(2, 17)_	0.004
	Sampling method * Field	0.0502	1.41_(2, 17)_	0.343
	Sampling method * Location	0.3738	10.52_(2, 17)_	0.025
	Field * Location	0.1012	2.85_(4, 17)_	0.167
Aphid abundance ^ in wheat	Sampling method	3.1546	8.00_(1, 14)_	0.066
	Field	0.0047	0.01_(2, 14)_	0.988
	Location (field vs. shelterbelt vs. refuge)	0.9916	2.52_(2, 14)_	0.228
	Sampling method * Field	0.0013	0.00_(2, 14)_	0.997
	Field * Location	0.0969	0.25_(4, 14)_	0.896
Proportion of alates in canola	Sampling method	0.0679	0.62_(1, 17)_	0.476
	Field	0.0228	0.21_(2, 17)_	0.820
	Location (field vs. shelterbelt vs. refuge)	0.0636	0.58_(2, 17)_	0.601
	Sampling method * Field	0.1141	1.04_(2, 17)_	0.433
	Sampling method * Location	0.0008	0.01_(2, 17)_	0.993
	Field * Location	0.0509	0.46_(4, 17)_	0.763
Proportion of alates in wheat	Sampling method	0.0053	0.26_(1, 14)_	0.648
	Field	0.0312	1.50_(2, 14)_	0.353
	Location (field vs. shelterbelt vs. refuge)	0.2707	13.05_(2, 14)_	0.033
	Sampling method * Field	0.0624	3.01_(2, 14)_	0.192
	Field * Location	0.0220	1.06_(4, 14)_	0.500
Mummy abundance ^ in canola	Field	0.0006	0.00_(2, 8)_	0.997
	Location (field vs. shelterbelt vs. refuge)	0.6275	1035.72_(2, 8)_	<0.001
Mummy abundance ^ in wheat	Field	0.0015	0.81_(2, 8)_	0.489
	Location (field vs. shelterbelt vs. refuge)	0.0023	1.41_(2, 8)_	0.315
Aphid parasitoid abundance ^ in canola	Sampling method	0.2565	9.98_(2, 26)_	0.007
	Field	0.0547	2.13_(2, 26)_	0.181
	Location (field vs. shelterbelt vs. refuge)	1.2703	49.42_(2, 26)_	<0.001
	Sampling method * Field	0.0257	1.00_(4, 26)_	0.462
	Sampling method * Location	0.0587	2.28_(4, 26)_	0.149
	Field * Location	0.0589	2.29_(4, 26)_	0.148
Aphid parasitoid abundance ^ in wheat	Sampling method	0.1152	22.21_(2, 26)_	0.001
	Field	0.0068	1.30_(2, 26)_	0.324
	Location (field vs. shelterbelt vs. refuge)	0.0093	1.79_(2, 26)_	0.228
	Sampling method * Field	0.0072	1.39_(4, 26)_	0.321
	Sampling method * Location	0.0148	2.84_(4, 26)_	0.097
	Field * Location	0.0059	1.14_(4, 26)_	0.405

^ Abundance logged using (log(x + 1)), * Interaction between factors. Significant results are displayed in bold.

**Table 4:** Results of MRPP analyses (A statistic, probability) on aphid parasitoid species composition.

Data Analysed	Crop			
	Canola		Wheat	
	A	*p*	A	*p*
**Species composition of aphid parasitoids across fields**				
(a) pan trapped 2017	−0.140	0.689	−0.129	0.566
(b) directly sampled 2018	−0.206	0.800	-	-
(c) vacuum sampled 2018	−0.083	0.954	0.038	0.283
(d) reared 2018	−0.230	0.915	−0.094	0.837
**Species composition of aphid parasitoids of field versus refuge**				
(a) pan trapped 2017	0.051	**0.013**	0.034	0.328
(b) directly sampled 2018	0.619	**0.042**	-	-
(c) vacuum sampled 2018	0.249	**0.004**	0.092	0.100
(d) reared 2018	0.684	**0.017**	0.068	0.263
**Species composition of aphid parasitoids across crop growth stages**				
(a) pan trapped 2017	−0.290	0.752	−0.175	0.842
(b) directly sampled 2018	0.025	0.324	0.018	0.277
(c) vacuum sampled 2018	0.057	0.061	0.191	**0.001**
(d) reared 2018	0.019	0.300	0.028	0.184

Significant results are displayed in bold.

## Data Availability

The data presented in this study are available on request from the corresponding author.

## Supplementary Materials

The following are available online at https://www.mdpi.com/2075-4450/12/1/44/s1, Figure S1: Population trends of primary and secondary parasitoids directly (a) and vacuum (b) sampled in canola and directly (c) and vacuum (d) sampled in wheat in 2018; Figure S2: Population trends of logged parasitoids (log(x + 1)) directly sampled from within canola paddocks in 2018 (Inset: pie charts showing wasp species composition pertaining to each respective visit); Figure S3: Population trends of logged parasitoids vacuum sampled (log(x + 1)) from (a) within canola paddocks, (b) at the edge of canola paddocks, (c) from within wheat paddocks, and (d) at the edge of wheat paddocks (d) in 2018; Figure S4: Population trends of logged parasitoids (log(x + 1)) reared from within canola paddocks (a), at the edge of canola paddocks (b), from within wheat paddocks (c), and at the edge of wheat paddocks (d) in 2018; Figure S5: The aphid population trends collected directly in (a) canola and (b) wheat, and by vacuum sampling in (c) canola and (d) wheat, for different distances into the paddocks in 2018; Figure S6: The aphid species directly sampled within (a) and at the edge of (b) canola paddocks, and within (c) and at the edge of (d) wheat paddocks in 2018.
