# Supplementary material for: Hymenopteran Parasitoids of Aphid Pests within Australian Grain Production Landscapes
Source: Insects. 2021 Jan 8;12(1):44. doi: 10.3390/insects12010044 (PMC7827963; doi:10.3390/insects12010044)
Supplement: Supplementary file 1 [file insects-12-00044-s001.pdf]

Supplementary material

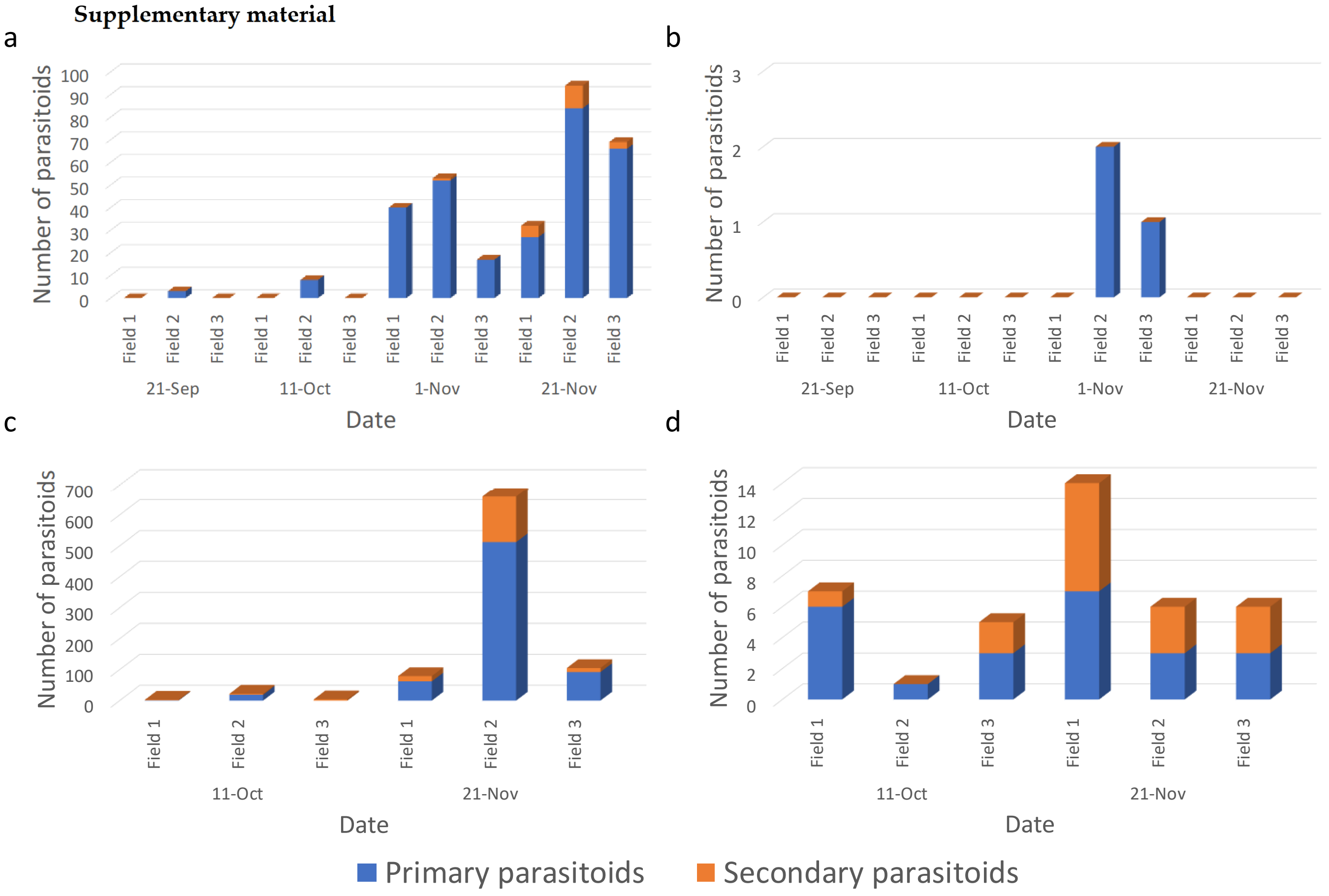

Figure S1: Population trends of primary and secondary parasitoids directly (a) and vacuum (b) sampled in canola and directly (c) and vacuum (d) sampled in wheat in 2018.

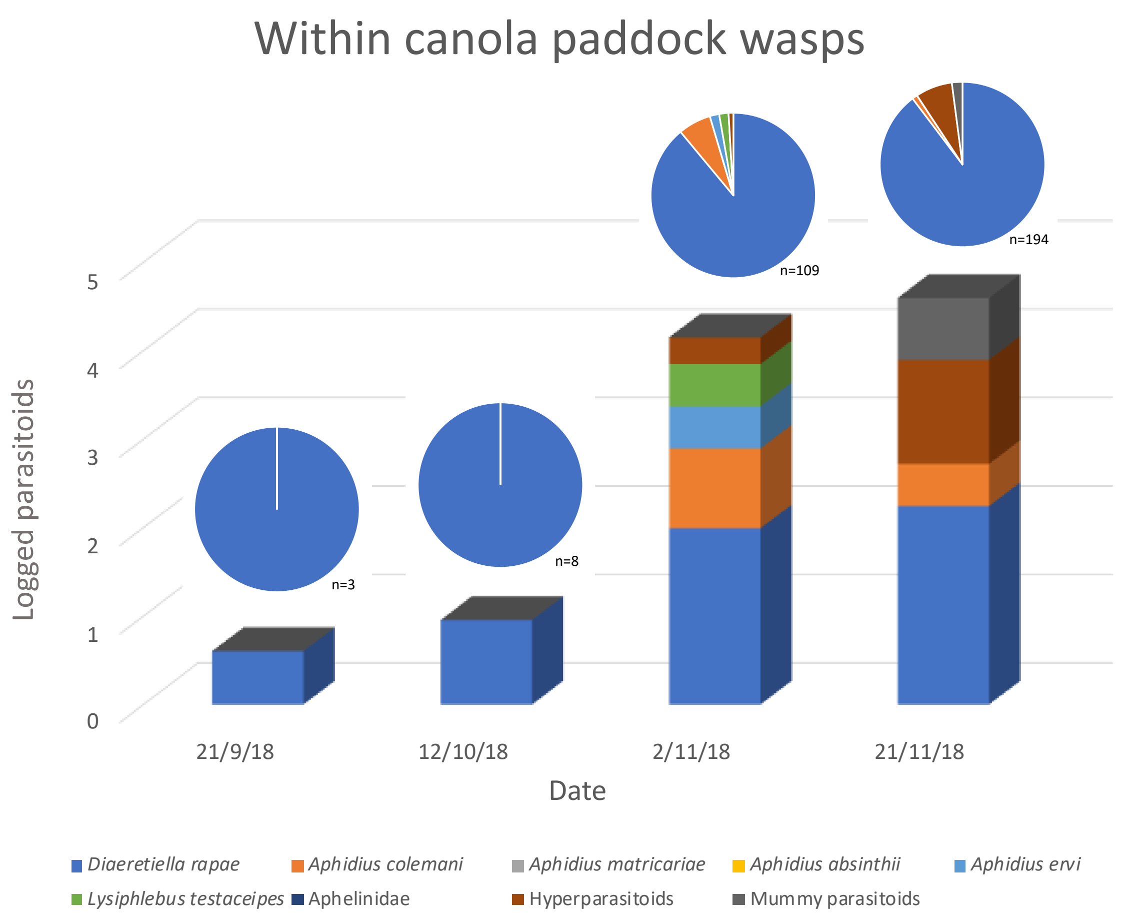

Figure S2: Population trends of logged parasitoids (log(x+1)) directly sampled from within canola paddocks in 2018 [Inset: pie charts showing wasp species composition pertaining to each respective visit].

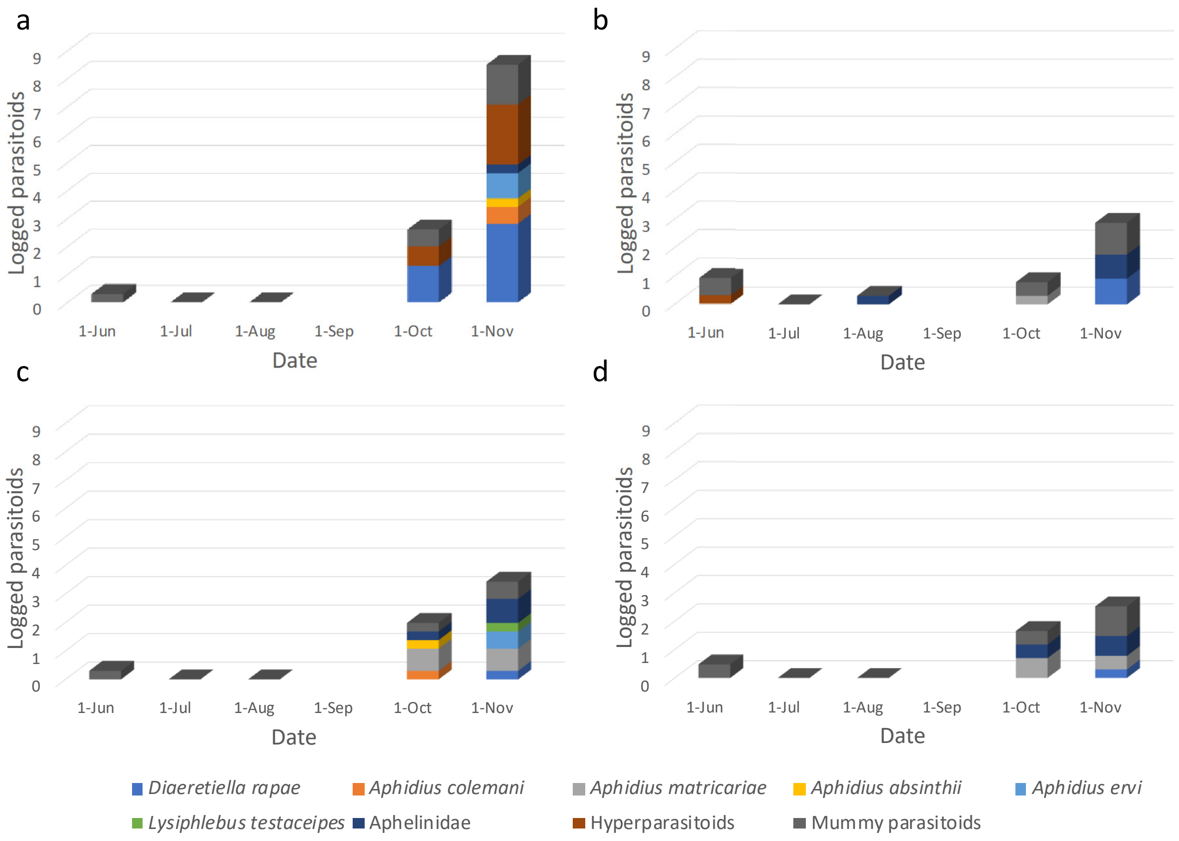

Figure S3: Population trends of logged parasitoids vacuum sampled (log(x+1)) from (a) within canola paddocks, (b) at the edge of canola paddocks, (c) from within wheat paddocks, and (d) at the edge of wheat paddocks (d) in 2018.

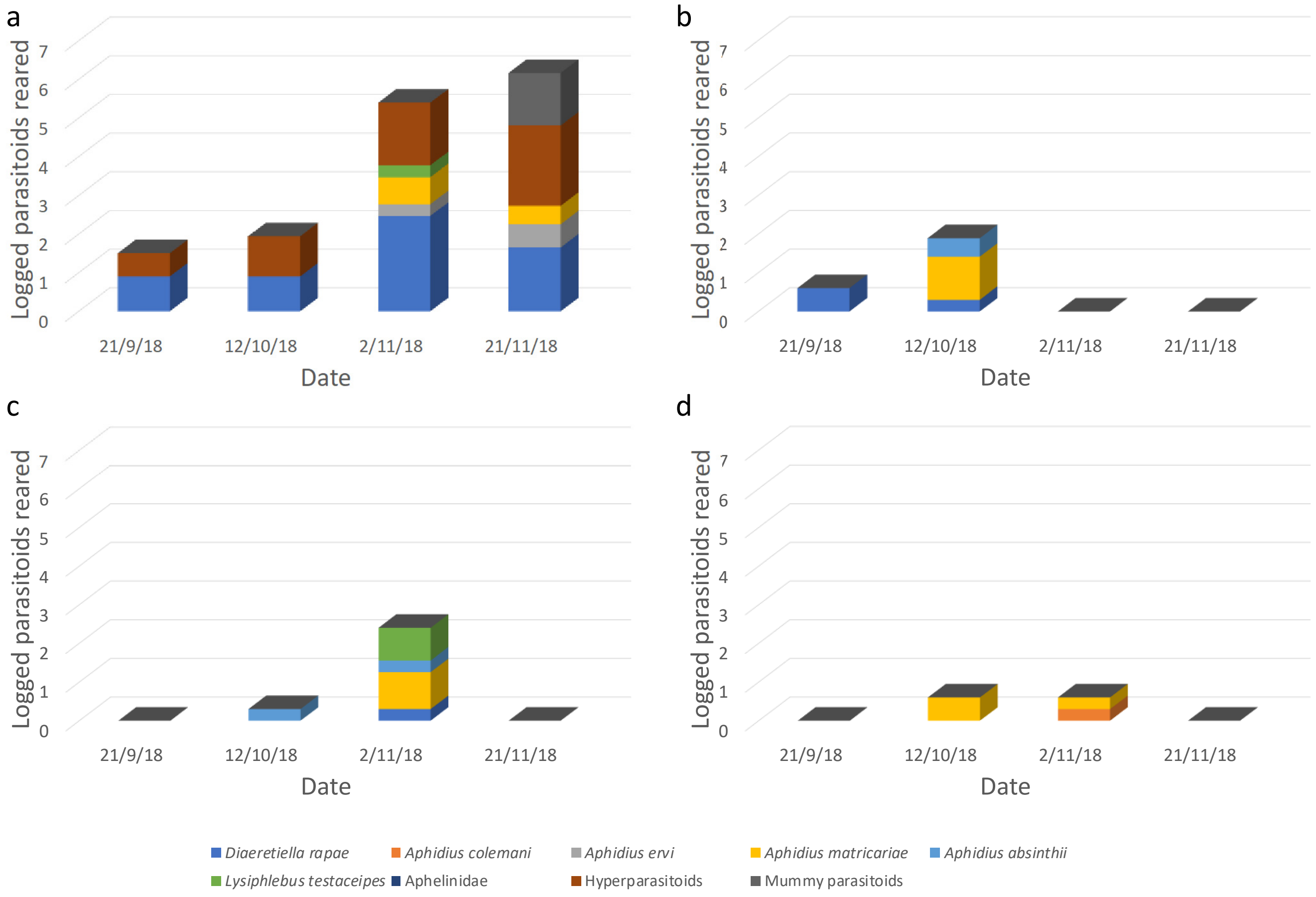

Figure S4: Population trends of logged parasitoids (log(x+1)) reared from within canola paddocks (a), at the edge of canola paddocks (b), from within wheat paddocks (c), and at the edge of wheat paddocks (d) in 2018.

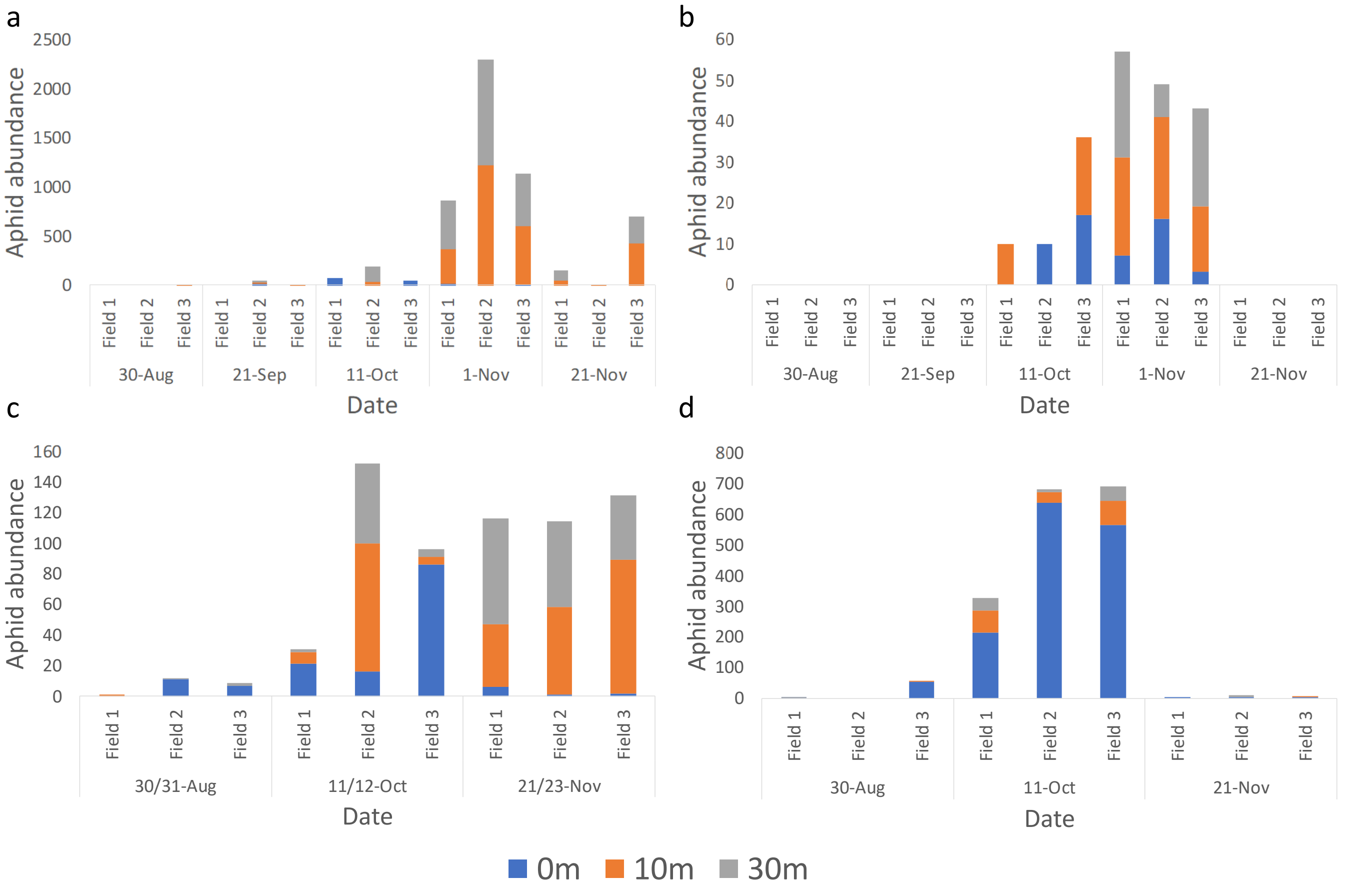

Figure S5: The aphid population trends collected directly in (a) canola and (b) wheat, and by vacuum sampling in (c) canola and (d) wheat, for different distances into the paddocks in 2018.

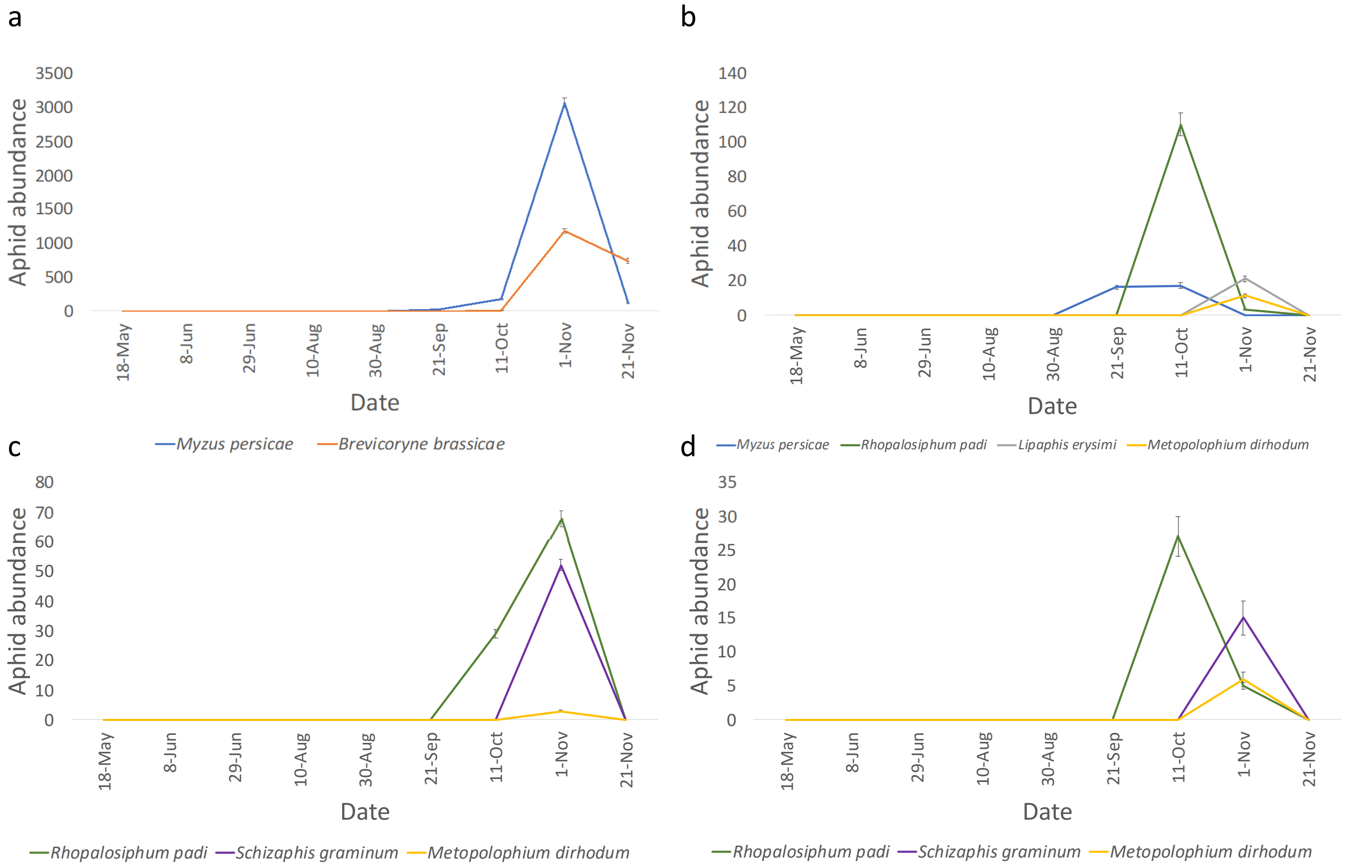

Figure S6: The aphid species directly sampled within (a) and at the edge of (b) canola paddocks, and within (c) and at the edge of (d) wheat paddocks in 2018.
